# Moringa oleifera potential for the treatment and prevention of COVID-19 involving molecular interaction, antioxidant properties and kinetic mechanism

**DOI:** 10.1371/journal.pone.0337904

**Published:** 2025-12-03

**Authors:** Mamoudou Hamadou, Christian Bernard Bakwo Bassogog, Fookalbo Vagai Obadias, Abras Djaffi Assaly, Martin Alain Mune Mune

**Affiliations:** 1 Department of Biological Sciences, Biochemistry, Bioinformatics, and Bioactive Compounds for Health Promotion, Research Unit, Faculty of Science, University of Maroua, Maroua, Cameroon; 2 Department of Chemistry, COMSATS University Islamabad, Islamabad, Pakistan; 3 Faculty of Science, University of Yaoundé I, Yaoundé, Cameroon; University of Mashreq, IRAQ

## Abstract

The COVID-19 pandemic, caused by the emergence of the SARS-CoV-2 virus in 2019, has led to a global state of emergency declared by the World Health Organization (WHO). The search for effective therapies against the virus is ongoing, and traditional medicine is emerging as a promising alternative, as many plants with known antiviral potential have been utilized during the pandemic. In this study, we investigated the inhibition of cysteine proteases (Mpro, PLpro, and papain) by bioactive compounds from *Moringa oleifera* leaves, with the ultimate goal of developing a novel treatment. We conducted a virtual screening based on the structure of the Mpro and PLpro enzymatic targets of SARS-CoV-2, for the selection of compounds with higher inhibitory potential. We then identified 14 bioactive compounds with high binding energy against Mpro and PLpro using AutoDock. Gamma-sitosterol was particularly promising since it showed higher interaction energy with Mpro (−8.6Kcal/mol) and PLpro (−7.4Kcal/mol). Phytochemicals in *Moringa oleifera* leaves were extracted using aqueous and hydromethanolic solvent system. It was found that polyphenols (4.20–5.01 mg GAE/g DM) and flavonoids (95.86–135.41 mg QE/ g DM) were the major constituents in the extracts. Antioxidant activity in the extracts, particularly DPPH scavenging activity (63.46–123.58 mg TE/g DM) and FRAP activity (48.14–49.66 mg TE/g DM), was high suggesting a potential for the prevention of oxidation and inflammation. Global and multiple alignment were conducted between Mpro, PLpro and papain, and results showed high conservation of the amino acid residues in the active site of the enzymes, suggesting a similar molecular catalysis mechanism. The extracts showed inhibitory activity against papain with the IC_50_ of 7.5 mg/mL for the hydromethanolic extract and 12.5 mg/mL for the aqueous extract. It was found in kinetic studies that hydromethanolic extract was a competitive inhibitor of papain, while aqueous extract was a hyperbolic non-competitive inhibitor, suggesting the presence of compounds with different interaction profiles to the enzymes. This study demonstrates that *Moringa oleifera* leaf extracts have substantial antioxidant activity and inhibit the model cysteine protease papain *in vitro*. These data, together with *in silico* docking against Mpro and PLpro, identify candidate phytochemicals that merit further evaluation; however, papain inhibition is a preliminary biochemical filter and does not by itself establish antiviral activity against SARS-CoV-2.

## Introduction

Coronaviruses are enveloped, positive-sense RNA viruses that infect a wide range of vertebrate hosts [1,2]. The emergence of severe acute respiratory syndrome coronavirus 2 (SARS-CoV-2), the etiologic agent of coronavirus disease 2019 (COVID-19), precipitated a global public-health crisis and highlighted the urgent need for safe, effective, and widely accessible antiviral therapies [3–6]. Antiviral drug discovery against SARS-CoV-2 has therefore prioritized essential viral enzymes whose inhibition blocks viral replication and pathogenesis [7–9].

Two cysteine proteases encoded by SARS-CoV-2, the main protease (Mpro, also known as 3C-like protease, 3CLpro) and the papain-like protease (PLpro), mediate proteolytic processing of the viral polyprotein to produce functional nonstructural proteins required for assembly of the replication–transcription complex [4,7]. In addition to polyprotein processing, PLpro exerts deubiquitinating and de-ISGylating activities (removal of the interferon-stimulated gene 15 protein, ISG15) that attenuate host innate immune signaling and favor viral persistence [7]. Because both Mpro and PLpro use an active Cys–His catalytic dyad/triad and are essential for viral replication and immune evasion, they represent high-value targets for small-molecule and natural-product inhibitors.

Natural products remain a prolific source of bioactive scaffolds for infectious-disease therapeutics, and ethnopharmacological leads are especially attractive when rapid, scalable screening is required [10–13]. *Moringa oleifera* is a multipurpose medicinal and nutritional plant whose leaves are rich in polyphenols, flavonoids, and other small molecules with reported anti-inflammatory and antioxidant properties [4,8,14,15]. Its use in traditional medicine to alleviate respiratory and inflammatory symptoms, combined with its broad phytochemical diversity [16–18], make *M. oleifera* leaf extracts a plausible source of protease-inhibitory compounds warranting systematic investigation.

Effective antiviral discovery pipelines integrate target-directed computational screening with biochemical validation. Molecular docking and related *in silico* approaches permit rapid prioritization of candidate ligands for SARS-CoV-2 Mpro and PLpro by predicting binding modes and relative affinities at atomic resolution [4,13,19]. Complementary *in vitro* biochemical assays are often required to confirm actual inhibitory activity against protease catalysis. However, access to recombinant viral proteases or cell-based viral assays usually takes time to set up. It is then important to develop strategies for evaluating viral proteases using available enzymes.

To bridge this practical gap while maintaining mechanistic relevance, we adopted a two-tiered strategy that combines molecular docking against the SARS-CoV-2 proteases with biochemical inhibition assays using papain (the archetypal plant cysteine protease). Papain (from *Carica papaya*) is a well-characterized member of clan CA (family C1) of cysteine proteases and shares fundamental mechanistic features with viral cysteine proteases, notably a catalytic cysteine and histidine that mediate nucleophilic attack on peptide bonds [20,21]. Although papain is structurally distinct from Mpro and PLpro and therefore cannot substitute for them in definitive antiviral testing, its conserved catalytic chemistry and commercial availability make it a pragmatic surrogate for preliminary biochemical screening of cysteine-targeting inhibitors. In this integrated design, *in silico* docking directly interrogates SARS-CoV-2 Mpro and PLpro to identify phytochemical ligands with favorable predicted interactions, while papain inhibition assays experimentally assess whether the same extracts or compounds possess cysteine protease inhibitory activity in solution. The concordance between docking predictions for viral proteases and observed inhibition of papain strengthens confidence in candidates for subsequent testing against recombinant viral proteases and cell-based antiviral assays.

Accordingly, the objective of the present study was to evaluate aqueous and hydromethanolic extracts of *Moringa oleifera* leaves for their potential to inhibit SARS-CoV-2 cysteine proteases by (i) performing molecular docking of identified phytochemicals against SARS-CoV-2 Mpro and PLpro to predict binding interactions, and (ii) measuring biochemical inhibition of papain as a surrogate cysteine protease. This combined computational–experimental strategy provides a cost-effective and mechanistically grounded screening pipeline that is especially applicable in laboratories with limited access to viral reagents. We emphasize that papain assays are an initial biochemical filter and that promising candidates identified here will require follow-up validation using recombinant viral proteases and cell-based infection models to establish antiviral efficacy and specificity.

## Materials and methods

### Identification and characterization of phytochemical compounds in *Moringa oleifera*

Phytochemical compounds were identified and retrieved by their chemical names from PubChem database [13,22]. Following selection, these compounds were filtered using available filters to restrict results to phytochemical compounds. For the post-filtration, the compounds were selected and characterized using a 3D molecular modeling tool available on PubChem.

### Preparation of ligands for docking study

Identified phytochemical compounds from *Moringa oleifera* were prepared for molecular docking simulation. 3D structures were downloaded from PubChem in sdf format. Subsequently, ligands were converted to mol2 format, and energy minimization was performed using Avogadro. All ligands’ structures were optimized using Avogadro. Following optimization, water elimination, addition of missing atoms, polar hydrogen addition, and Kollman charge assignment were executed [13,23,24].

### Preparation of COVID-19 proteases for docking study

3D crystal structures of SARS-CoV-2 spike glycoprotein (PDB ID: 6LU7 for Mpro and PDB ID: 7JRN for PLpro) were obtained from the RCSB database. For molecular docking studies, complete protein structures in PDB format were selected. All 3D protein structures were prepared using AutoDockVina 1.5.7.

### Molecular docking simulations

#### Docking parameters and grid generation.

We performed molecular docking simulations with AutoDock Vina (v1.5.7) [23,24], employing the Lamarkian Genetic Algorithm and multithreading. The search grid for each enzyme target was centered on its crystallographic active site, with box dimensions defined using a co-crystallized reference inhibitor: for Mpro (PDB: 6LU7), a 35.59 × 45.78 × 40.19 Å^3^ box centered on (20.9810, 15.4614, 60.8432) involved the N3 inhibitor site; for PLpro (PDB: 7JRN), a 25.00^3^ Å^3^ box centered on (15.4870, 54.4870, 6.5190) included the GRL0617 site. An exhaustiveness value of 8 balanced thoroughness with computational efficiency. We executed 20 independent docking runs per ligand, clustered the output poses, and selected the conformation with the most favorable calculated binding free energy (ΔG) for subsequent analysis.

#### Docking protocol validation.

**Fig 1:**
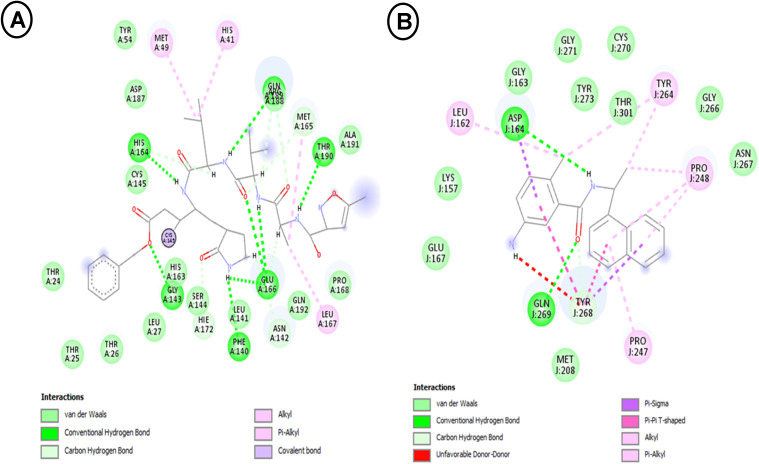
A-B. Molecular interactions between (A) N_3_inhibitor –Mpro and (B) GRL0617- PLpro.

We validated the docking protocol by redocking each enzyme’s native co-crystallized ligand (N3 for Mpro, GRL0617 for PLpro) into its respective binding site (Fig 1A-B). The root-mean-square deviation (RMSD) between the best-docked position and the empirically established crystallographic pose was less than 2.0 Å for both complexes. This result shows that the method is accurate because it can reproduce known binding geometries. These confirmed native ligands functioned as positive controls for comparative binding affinity assessments during the investigation.

### Investigating the similarity between PLpro/Mpro proteases and papain

#### Global alignment of COVID-19 proteases in EMBOSS.

The primary structure of papain was retrieved from the UniProtKB database (UniProtKB ID: P00784) in FASTA format [25]. The FASTA file was inserted into the Needle algorithm (in the Sequence section) in EMBOSS, and then analyzed. The aligned sequences were recorded by running the ‘OUTFILE’ option and saving the output to a specified file.

#### Multiple alignment of COVID-19 proteases in clustal omega.

The FASTA sequences of two COVID-19 proteases and papain were analyzed using the online interface on the EMBL-EBI website (https://www.ebi.ac.uk/Tools/msa/clustalo/) by the Clustal Omega program [26].

### *In silico* analysis of promising candidates’ pharmacokinetics

Pharmacokinetic analysis of promising compounds was using the AI Drug Lab ADMET online tool (https://ai-druglab.smu.edu/admet) [27].

### Ethics approval

Ethics approval was not necessary since humans and animals were not included in the study.

### *In vitro* analysis

#### Plant material.

The plant material consisted of leaves of *Moringa oleifera* collected from a garden in the city of Maroua at geographical coordinates 10°587438 North and 14°280158 East. The leaves were harvested and dried in the shade.

#### Sample preparation: production of *Moringa oleifera*leaf powder.

The *Moringa oleifera* leaves were cleaned, ground, and sieved (Fisher Scientific, France) through a 250 μm mesh. The resulting powder was weighed and stored in black polyethylene bags at 4°C.

#### Extraction of phenolic compounds.

The extraction of phenolic compounds was carried out using a methanol/water mixture (70/30) for the hydro-methanolic extract, and distilled water (100%) for the aqueous extract [28,29].

#### Hydro-methanolic extraction.

Twenty grams of *Moringa oleifera* leaf powder were weighed using a balance (Precisa 12000D CSC) and then immersed in 320 mL of methanol and 80 mL of distilled water for 24 hours at room temperature. The mixture was filtered through filter paper. The filtrate was dried under reduced pressure (69.5°C) using a rotary evaporator (Rota-vapor) for 2 hours. The resulting solution was then placed in an oven at 50°C for 48 hours. The obtained extract was stored in a polyethylene bag at 4°C.

#### Aqueous extraction.

Twenty grams of *Moringa oleifera* leaf powder were weighed using a balance (Precisa 12000D CSC) and then immersed in 400 mL of distilled water for 24 hours at room temperature. The mixture was filtered through filter paper. The obtained filtrate was placed in a rotary evaporator at 50°C for 48 hours. The resulting extract was stored in a polyethylene bag at 4°C.

#### Phytochemical analysis.

A mass of 0.515g of the aqueous extract and 0.557g of the hydro-methanolic extract were separately diluted in 10 mL of distilled water. The mixture was stirred using a magnetic stirrer at room temperature.

***Total polyphenol content*** [11,30,31]: One milliliter of Folin-Ciocalteu reagent (a mixture of phosphotungstic acid and phosphomolybdic acid) was added to 0.3 mL of the sample. Then, 3 mL of 7.5% sodium bicarbonate was added. The mixture was incubated in the dark for 1 hour. The optical density was read at 750 nm. The concentrations were determined by referring to the calibration curve obtained using gallic acid. The results were expressed in gallic acid equivalents (mg GAE/g dry matter).

***Flavonoid content*** [11,31,32]: One and a half milliliters of aluminum trichloride (AlCl_3_) were added to 3 mL of the sample and stirred for 1 minute. Then, 6 drops of 1% acetic acid were added, and the mixture was stirred. The optical density was read at 430 nm. The concentrations were determined by referring to the calibration curve obtained using quercetin. The results were expressed in quercetin equivalents (mg QE/g dry matter).

***Tannin content*** [11,31,32]: Four and a half milliliters of vanillin were added to 1.5 mL of the sample and incubated at 30°C in the dark for 5 minutes. The optical density was read at 500 nm.The concentrations were determined by referring to the calibration curve obtained using catechin. The results were expressed in catechin equivalents (mg CE/g dry matter).

#### *In vitro* evaluation of antioxidant properties of *Moringa oleifera’s* extract.

***DPPH antiradical activity*** [11,29,31]: A freshly prepared DPPH solution (4.5 mL) was added to 1.5 mL of hydrolysate (aqueous and methanolic extracts) in a test tube, followed by mixing. The mixture was incubated in the dark for 15 minutes at ambient temperature, and the optical density was measured at 517 nm. Trolox was prepared at concentrations of 0, 25, 50, 75, 100, and 125 μg/mL as a reference, and a calibration curve was plotted. The DPPH activity was expressed as Trolox equivalents (mg TE/g DM).

***Ferric-reducing antioxidant power (FRAP) assay*** [31,33]: A solution of Fe(III)-TPTZ (4.5 mL) was added to 1.5 mL of hydrolysate in a test tube, followed by mixing and incubation for 30 minutes. The optical density was measured at 593 nm. Trolox was prepared at concentrations of 0, 25, 50, 75, 100, and 125 μM as a reference, and a calibration curve was plotted. The FRAP activity was expressed as Trolox equivalents (mg TE/g DM).

#### IC_50_ and inhibition kinetics of papain.

***Initial velocity measurement:*** The papain solution was prepared by mixing papain powder (Sigma Aldrich) with Tris-HCl buffer pH 7.6 to produce a solution at 10U/mL. Albumin (Sigma Aldrich) was selected as protein substrate for papain and prepared the concentration 10 mg/mL in distilled water. The enzymatic reaction was initiated by mixing 50 µL enzyme solution, 50 µL distilled water and100 μL of albumin. Reaction was stopped after 0, 5, 10-, 15-, 20-, and 25-minutes incubation at 60ºC, by heating at 100ºC for 5 min. Subsequently, 100 μL of ninhydrin solution was added and the reaction was preceded as described by [34,35]. Absorbance was measured at 590nm. Results found here were important for the inhibition experiments.

***Inhibition of papain:*** Aqueous and methanolic extracts of *Moringa oleifera* leaves (inhibitors) were prepared at different concentrations from 0 mg/mL to 50 mg/mL in distilled water. Pre-incubation was initiated by mixing 50 µL of enzyme solution with 50 µL inhibitor solution, and hold the mixture at 60ºC for 10 min. Then, a volume of 100 µL of the substrate was added and the reaction proceeded at 60ºC for 15 min. The solution was heated for 10 min in boiling water and free NH_2_ was quantified using the ninhydrin reaction. The inhibitory activity was calculated by the following formula (Equation 1):


$$\text{Inhibition activity}(\%)=(\text{1}-(\frac{A_{S}-A_{\text{0}}}{A_{C}}))\times \text{1}\text{0}\text{0}$$ (1)

Where A_S_ represents the absorbance of the sample, A_c_ is the absorbance of the control without the inhibitor, and A_0_ is the absorbance of the sample without the enzyme. The half-maximum inhibition concentration (IC_50_) was calculated using nonlinear regression.

***Kinetic analysis of the inhibition:*** For this study the inhibitor was prepared at 0, 30 and 50 mg/ml. and the substrate at 2 mg/mL, 4 mg/mL, 8 mg/mL, and 10 mg/mL and the reaction proceeded as described above. The initial velocity was calculated in ΔOD/min. The values of initial velocity at different substrate concentrations and at each inhibitor concentration were plotted (Michaelis–Menten plot), as well as the double reciprocal plot at each inhibitor concentration (Lineweaver–Burk plot).

The Michaelis–Menten equation for competitive inhibition (Equation 2) is given as follows [36]:


$$v=\frac{V_{\text{max}}\times S}{K_{\text{m}}\left(\text{1}+\frac{I}{Ki}\right)+S}=\frac{V_{\text{max}}^{app}\times S}{K_{\text{m}}^{app}+S}$$ (2)

And the corresponding Lineweaver–Burk equation is given as follows (Equation 3):


$$\frac{\text{1}}{v}=\frac{K_{\text{m}}}{V_{\text{max}}}\left(\text{1}+\frac{I}{Ki}\right)\frac{\text{1}}{S}+\frac{\text{1}}{V_{\text{max}}}$$ (3)

The Michaelis–Menten equation for hyperbolic non-competitive inhibition (Equation 4) is given as follows [36]:


$$v=\frac{V_{\text{max}}\left(\frac{\text{1}+I/K_{\text{I}}}{\text{1}+\beta I/K_{\text{I}}}\right)\times S}{K_{\text{m}}\left(\frac{\text{1}+I/Ki}{\text{1}+\beta I/K_{\text{I}}}\right)+S}=\frac{V_{\text{max}}^{app}\times S}{K_{\text{m}}^{app}+S}$$ (4)

And the corresponding Lineweaver–Burk equation is given as follows (Equation 4):


$$\frac{\text{1}}{v}=\frac{K_{\text{m}}}{V_{\text{max}}}\left(\frac{Ki+I}{K_{\text{I}}+\beta I}\right)\frac{\text{1}}{S}+\frac{\text{1}}{V_{\text{max}}}\left(\frac{K_{\text{I}}+I}{K_{\text{I}}+\beta I}\right)$$ (5)

where *v* is the initial velocity, *V*_max_ is the maximum initial reaction velocity, *I* is the concentration of the inhibitor, *S* is the concentration of the substrate, *K*_m_ is the Michaelis constant, Ki is the competitive inhibition constant and K_I_ is the inhibitor constant for the complex ESI, and β is the inhibitory coefficient.

### Statistical data analysis

All results are presented as mean ± standard error of the mean (SEM). Data analysis was performed using SPSS software (version 25). One-way analysis of variance (ANOVA) was employed to compare means among groups, followed by Tukey’s post-hoc test to determine significant differences between individual groups. A significance level of α = 0.05 was used to determine statistical significance.

## Results and discussion

### Phytochemicals in *Moringa oleifera*

The results in Table 1 and S1 Fig provide a detailed profile of the phytochemicals found in *Moringa oleifera* leaves, highlighting their classifications, taxonomy, molecular formulas, and molecular masses. The table categorizes these compounds into various chemical families, revealing the diversity of bioactive substances present in *Moringa oleifera*. The phytochemical compounds in *Moringa oleifera* leaves display a diverse range of bioactivities (Table 1), contributing to the plant’s medicinal value. Each compound plays a significant role in the plant’s biological activity, supporting its use in traditional medicine for treating various health conditions and symptoms common to COVID-19 [16,17,37–42].

**Table 1:** Phytochemicals from *Moringa oleifera* leaves.

N°	Compounds	Classification	Taxonomy	Formula	Molecular mass
01	1, 2,3-Cyclopentanetriol	Belongs to the polyol family	Classified as trisubstituted cyclopentane	C_5_H_10_O_3_	118.13g/mol
02	2,6-Dihydroxybenzoic acid	Belongs to the class of phenolic acids	Family of carboxylic acids and more specifically in the subclass of phenolic acids	C_7_H_6_O_4_	154.12g/mol
03	Ethyl oleate	Classified as esters	Classified in the family of esters and more specifically in the subclass of fatty acid esters.	C_20_H_38_O_2_	310.5g/mol
04	Gamma-sitosterol	Belongs to the class of phytosterols	Classified in the sterol family	C_29_H_50_O	414.7g/mol
05	Hexadecanal	Aldehydes	Aldehydes and more specifically in the subclass of aliphatic aldehydes	C_16_H_32_O	240.42g/mol
06	Octadecanoic acid	Saturated fatty acid	Classified as a lipid	C_18_H_36_O_2_	284.5g/mol
07	Oleic acid	Belongs to the family of omega-9 fatty acids.	Classified in the family of fatty acids and more specifically in the subclass of monounsaturated fatty acids	C_18_H_34_O_2_	282.46g/mol
08	Phytol	Belongs to the terpene family	Classified in the terpene family and more specifically in the diterpene class	C_20_H_40_O	296.5g/mol
09	Pregn-5,7-diene-3-ol20-one	Belongs to the steroid class	Classified in the steroid family	C_21_H_30_O_2_	314.5g/mol
10	Quinic acid	Carboxylic acids	Carboxylic acids and more specifically it is classified in the subclass of hydroxycarboxylic acids	C_7_H_12_O_6_	192.17g/mol
11	Vitamin E	Fat-soluble vitamin	Belongs to the class of tocopherols and tocotrienols	C_27_H_46_O_2_	402.7g/mol
12	cis-vaccenic acid	Unsaturated fatty acid	Classified as a lipid	C_18_H_34_O_2_	282.5g/mol
13	L-galactose, 6-deoxy	Belongs to the aldohexose family	Classified as an aldehyde	C_6_H_12_O_5_	164.16 g/mol
14	n-Hexadecanoic acid	Saturated fatty acid	Classified as a lipid	C_16_H_32_O_2_	256.42g/mol

As a phenolic acid, 2,6-dihydroxybenzoic acid possesses strong antioxidant activity. Phenolic acids are well-documented for their ability to neutralize free radicals, which can prevent cellular damage. This property is crucial in the prevention of chronic diseases such as cancer, cardiovascular diseases, and diabetes. Additionally, phenolic acids have anti-inflammatory properties, potentially reducing inflammation and related conditions [38–41]. Phytosterols, such as gamma-sitosterol, are structurally similar to cholesterol and are known to lower blood cholesterol levels by inhibiting its absorption in the intestines. This helps in reducing the risk of cardiovascular diseases. Additionally, gamma-sitosterol exhibits anti-inflammatory and anticancer properties, making it beneficial for overall health [16,39–43].

The phytochemicals found in *Moringa oleifera* leaves are rich in bioactive compounds with diverse health benefits. These compounds, such as phenolic acids, fatty acids, terpenes, and vitamins, exhibit antioxidant, anti-inflammatory, antimicrobial, and cholesterol-lowering properties [16,39–45].This phytochemical profile supports *Moringa oleifera*’s traditional use in promoting cardiovascular health, managing diabetes, enhancing immune function, and protecting against chronic diseases, positioning it as a valuable source of natural health-promoting compounds [46–48].

### Binding energy *Moringa oleifera* phytochemicals and SARS-CoV-2 proteases

**Table 2** presents the binding energies (in kcal/mol) of 14 compounds derived from *Moringa oleifera*, as evaluated against two key proteases of SARS-CoV-2: PLpro (papain-like protease) and Mpro (main protease). A lower binding energy (more negative value) indicates a stronger and potentially more effective interaction, suggesting that the compound may inhibit the protease’s function, which is critical for the viral life cycle.

**Table 2:** Binding score of *Moringa oleifera* compounds.

Ligand		Binding energy (kcal/mol)	
		PLpro	Mpro
*Moringa oleifera’s* compounds	1-2-3-Cyclopentanetriol	−6.4	−6.4
	2-6-Dihydroxybenzoic-acid	−7.3	−7.4
	Ethyl-oleate	−7.8	−6.1
	Gamma-Sitosterol	−7.4	−8.6
	Hexadecanal	−6.4	−5.8
	Octadecanoic-acid	−6.8	−6.0
	Oleic-acid	−6.7	−6.0
	Phytol	−6.9	−6.9
	Pregn-5–7-diene-3-ol20-one	−7.2	−8.4
	Quinicacid	−6.5	−7.4
	VitaminE	−6.5	−8.2
	Cis-Vaccenic-acid	−6.9	−6.6
	L-galactose-6-deoxy	−6.1	−7.0
	n-hexadecanoicacid	−6.9	−6.2
Standards	N_3_ inhibitor	/	8.2
	GRL0617	−7.3	/

2,6-Dihydroxybenzoic acid demonstrated stronger binding than 1,2,3-cyclopentanetriol, with binding energies of −7.3 kcal/mol for PLpro and −7.4 kcal/mol for Mpro (**Table 2**). The capacity of this compound to engage both proteases at comparable levels suggests that it may play a role in protease inhibition in association with antioxidant properties, and potentially contributing to viral suppression. Furthermore, ethyl oleate exhibits the lowest binding energies, at −7.8 kcal/mol for PLpro and −6.1 kcal/mol for Mpro (**Table 2**). This suggests that ethyl oleate is one of the most promising inhibitors into this set of compounds, particularly given its affinity for both proteases [49]. The strong binding of ethyl oleate (−7.8 kcal/mol) and Gamma-sitosterol (−7.4 kcal/mol) to PLpro is noteworthy, as these affinities are superior to or comparable with the well-established PLpro inhibitor GRL0617 (−7.3 kcal/mol). Its strong binding indicates the potential for blocking the function of these enzymes, which are critical for viral replication.

Gamma-sitosterol displays a markedly robust interaction with Mpro, exhibiting the highest binding energy in the table at −8.6 kcal/mol, and also demonstrates a notable affinity for PLpro, with a binding energy of −7.4 kcal/mol (**Table 2**). Crucially, the binding affinity of Gamma-sitosterol (−8.6 kcal/mol) exceeds that of the potent Mpro N3 inhibitor (−8.2 kcal/mol), indicating that this phytosterol is a highly promising lead structure for Mpro inhibition. Gamma-sitosterol, a phytosterol, is known for its cholesterol-lowering effects. However, the binding energies observed suggest a potential for interfering with viral proteases, which could be harnessed in antiviral therapies. Moreover, pregn-5,7-diene-3-ol-20-one exhibits notable binding affinity with both proteases, with binding energies of −7.2 kcal/mol for PLpro and −8.4 kcal/mol for Mpro (**Table 2**). The robust interaction with Mpro indicates the potential for therapeutic utility in the inhibition of viral pathogens, particularly in light of the steroid capacity to modulate immune responses.

The binding energies of −6.5 kcal/mol for PLpro and −7.4 kcal/mol for Mpro indicate a relatively strong interaction, particularly with Mpro (**Table 2**). Given its established role in antioxidant activity, quinic acid may serve to complement its potential antiviral effects by protecting cells from oxidative stress while inhibiting protease function. Furthermore, cis-vaccenic acid exhibited binding energies of −6.9 kcal/mol for PLpro and −6.6 kcal/mol for Mpro. The moderate interaction with both proteases indicates the potential for an additional antiviral role, in addition to the known benefits of lipid regulation [50,51].

Among the 14 compounds analyzed, ethyl oleate, gamma-sitosterol, pregn-5,7-diene-3-ol-20-one, and vitamin E appear as the most promising inhibitors based on their strong binding energies with both SARS-CoV-2 proteases, especially Mpro. These compounds could be explored further for their potential antiviral activity, particularly in drug design or therapeutic applications. The moderate binding energies of other compounds, such as oleic acid, phytol, and quinic acid, also suggest potential synergistic effects when used in combination with stronger inhibitors. Significantly, the affinity profiles of Gamma-sitosterol and pregn-5,7-diene-3-ol-20-one for Mpro surpass the predicted binding energy of the reference inhibitor (N3), providing high-level computational evidence that these Moringa compounds are strong candidates for further experimental investigation.

### Molecular interactions between SARS-CoV-2 proteases (Mpro and PLpro) and *M. oleifera* phytochemicals

**Fig 2:**
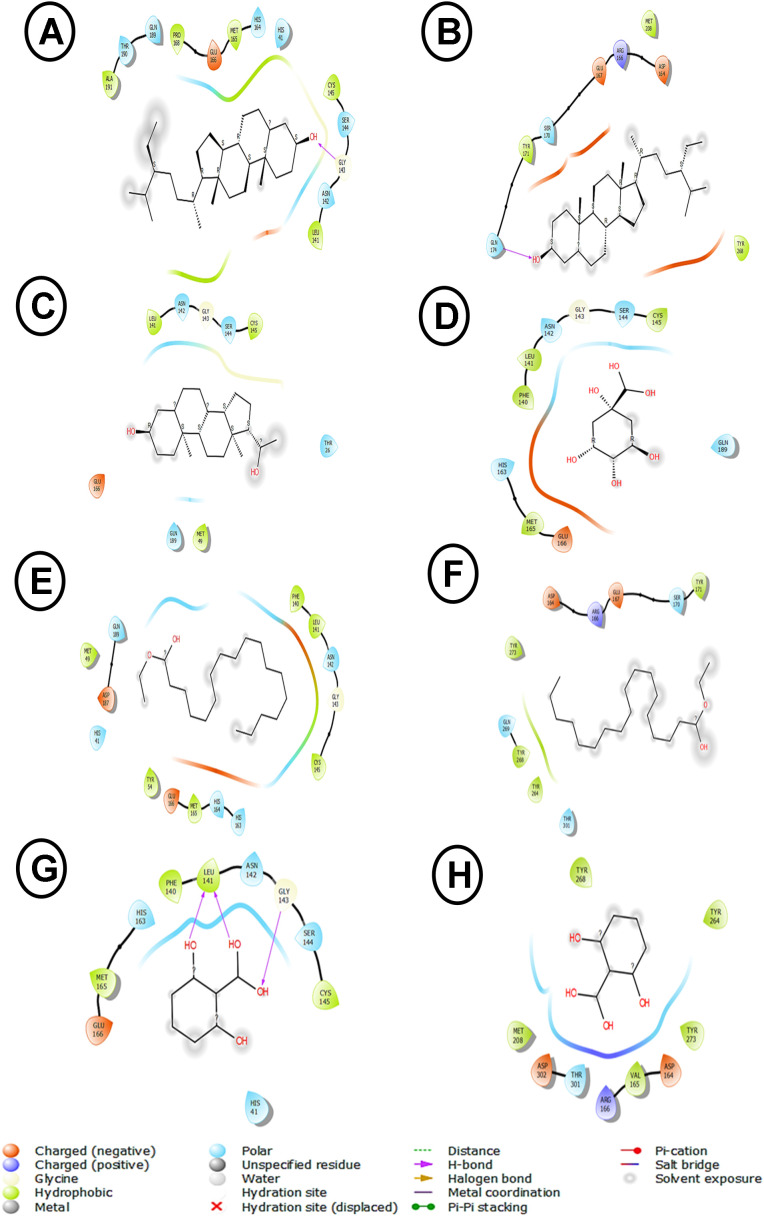
Molecular interactions between Moringa oleifera’s bioactive compounds and SARS-Cov-2 viral proteins (Mpro and PLpro). (A) γ-sitosterol-Mpro. (B) γ-sitosterol-PLpro. (C) pregn-5,7-diene-3-ol20-one – Mpro. (D) quinic acid – Mpro. (E) ethyl oleate–Mpro.(F) ethyl oleate–PLpro. (G) 2,6-dihydroxybenzoic acid – Mpro. (H) 2,6-dihydroxybenzoic acid – PLpro.

The molecular docking simulation presented in **Fig 2A** provides insights into the potential binding interactions between the phytochemical compound gamma-sitosterol and the Main Protease (Mpro) of SARS-CoV-2, the causative agent of COVID-19. Mpro is a key viral enzyme responsible for the processing of viral polyproteins and is considered a promising therapeutic target for the development of anti-COVID-19 agents [3,5,8,52].This process is dependent on the conserved Cys145/His41 catalytic dyad. The binding pose shows multiple stabilizing interactions, particularly notable are the conventional hydrogen bonds with Gly143 and Ser144 (**Fig 2A**), which are critical residues in the catalytic site of Mpro. The interaction with Gly143 and Ser144 is particularly significant as these residues form the oxy-anion hole responsible for stabilizing the transition state during substrate cleavage, thus providing a compelling mechanism of inhibition. The binding is further stabilized by van der Waals interactions with residues including His41, His163, His164, Met165, and Glu166, forming a complex network of non-covalent interactions. The presence of hydrophobic residues Leu141, Phe140, and Cys145 appears to create a favorable pocket for the sterol scaffold. These findings align with recent literature, particularly the study by Verma *et al.*[4], which identified natural compounds as a promising Mpro inhibitor. However, our docking results show additional interaction points, specifically with Arg188 and Gln189, which weren’t previously reported.

From the molecular docking visualization presented in **Fig 2B**, γ-sitosterol demonstrates notable interactions with SARS-CoV-2 PLpro (Papain-Like Protease), exhibiting both van der Waals interactions and conventional hydrogen bonding. The key binding interactions involve residues Gln-J:174, Ser-J:170, Arg-J:166, Asp-J:164, Glu-J:167, Met-J:208, Tyr-J:171, Thr-J:301, and Gln-J:269, with a distinctive Pi-Alkyl interaction with Tyr-J:268. These interactions suggest a stable binding configuration that could potentially inhibit PLpro activity. While gamma-sitosterol does not directly interact with the catalytic triad residues (Cys111, His272, Asp286), its placement within the S1 pocket, defined by the Pi-Alkyl interaction with Tyr-J:268, indicates a mechanism that sterically obstructs substrate access to the active site [4,7,53]. Recent literature supports these findings, as Majumder and Mandal [54] demonstrated that bioactive compounds can effectively bind to various SARS-CoV-2 proteins. The hydrogen bonding patterns observed in our docking results align with those reported by Chebaibi *et al*. [8], who found that plant-derived sterols show promising inhibitory potential against SARS-CoV-2 proteases. The significance of these results lies in the potential therapeutic application of γ-sitosterol as a naturally occurring compound with anti-SARS-CoV-2 properties. The observed binding pattern suggests that γ-sitosterol could interfere with PLpro’s catalytic activity, which is crucial for viral replication. Notably, the interaction with Tyr-268 through Pi-Alkyl bonding represents a potentially important binding feature not extensively discussed in previous studies [28,46,55,56]. This finding warrants further investigation through experimental validation and molecular dynamics simulations to confirm the stability and therapeutic potential of this interaction.

The molecular docking results illustrate the interaction between pregn-5,7-diene-3-ol20-one and the main protease (Mpro) of SARS-CoV-2(**Fig 2C**), providing insight into the antiviral potential of this steroid-like compound against COVID-19. The docking simulation shows that pregn-5,7-diene-3-ol20-one interacts with several key residues in the Mpro binding pocket. Notably, Cys145, His41, and Gly143 are involved in alkyl interactions (**Fig 2C**), while other residues such as Leu141, Thr25, and Gln189 engage through van der Waals forces. The involvement of Cys145 and His41 is particularly significant, as these residues are crucial to the catalytic activity of Mpro [4,19,54,57], making them common targets in drug design for SARS-CoV-2 protease inhibitors. However, the lack of polar interaction with the Cys145 side chain, which is often a feature of covalent or tightly binding reversible inhibitors, suggests the interaction is non-covalent and likely less potent than ideal Mpro ligands [53]. When compared to similar studies on other potential Mpro inhibitors, such as compounds derived from flavonoids and other natural products, pregn-5,7-diene-3-ol20-one shows a distinct binding profile. Previous research has shown that compounds like quercetin and baicalin interact with residues including Cys145 and His41 but also involve additional hydrogen bonds with Glu166, enhancing their binding stability and efficacy [3–5,54,58,59]. The lack of hydrogen bonding in pregn-5,7-diene-3-ol20-one’s interaction with Mpro may affect its binding affinity, suggesting it could be less effective in occupying and inhibiting the catalytic site. However, the alkyl interaction with Cys145 and His41 still implies potential inhibitory effects, particularly given the structural rigidity and stability of pregn-5,7-diene-3-ol20-one. This result indicates a potential for pregn-5,7-diene-3-ol20-one as an antiviral candidate, albeit possibly with lower affinity than other Mpro inhibitors unless optimized through structural modifications to enhance its interactions, such as introducing functional groups to facilitate hydrogen bonding with key residues like Glu166 [4,7,60].

Molecular docking analysis revealed significant interactions between quinic acid and SARS-CoV-2 Main Protease (Mpro) (**Fig 2D**). Visualization of the docking results showed multiple conventional hydrogen bonds with Ser-A:144 and Leu-A:141, crucial for stabilizing the ligand-protein complex. Furthermore, van der Waals interactions with His-A:172, His-A:164, His-A:163, Phe-A:140, Cys-A:145, Glu-A:166, Met-A:165, Gln-A:189, and Asn-A:142 residues characterized the binding pocket (**Fig 2D**), forming a comprehensive network of stabilizing interactions. These findings are consistent with recent research by Latha *et al*. [58], demonstrating favorable binding of naturally occurring compounds with multiple hydroxyl groups to Mpro’s catalytic site. Notably, our results revealed additional interactions with His-163 and His-164 not prominently featured in previous studies, suggesting a potentially unique binding mode. These results lie in quinic acid’s potential as a natural Mpro inhibitor [58,61,62]. Strong and specific binding, facilitated by multiple hydrogen bonds (notably with Ser-144 and Leu-141), could effectively inhibit protease activity essential for viral replication. The stable binding configuration, indicated by numerous van der Waals interactions, warrants further investigation through experimental validation to confirm interaction stability and therapeutic potential in COVID-19 treatment strategies [8,63]. Specifically, the conserved His-164 interaction merits further examination as a crucial component of the compound’s inhibitory mechanism.

The molecular docking analysis of ethyl oleate with the SARS-CoV-2 main protease (Mpro) (**Fig 2E**) was conducted to investigate potential inhibitory effects. The analysis revealed specific interactions between ethyl oleate and Mpro, indicating a distinct binding profile compared to established inhibitors. Ethyl oleate primarily engages in van der Waals interactions with amino acids Met49, Met165, Glu166, Cys145, and Asn142 within the active site of Mpro [4,64,65]. Additionally, a hydrogen bond is formed with Gln189, potentially contributing to the stabilization of the binding. Furthermore, alkyl and Pi-alkyl interactions were observed with His41 and Phe140 (**Fig 2E**), key residues in the catalytic site of Mpro. The interaction with His41, a crucial component of the catalytic dyad of Mpro, highlights the significance of ethyl oleate’s binding. The hydrophobic binding profile, characterized by alkyl and van der Waals forces, indicates that ethyl oleate functions as an allosteric inhibitor, likely inducing a conformational change that restricts Mpro’s flexibility instead of directly occupying the catalytic pocket [13,53]. In comparison to other natural compounds studied for Mpro inhibition, such as flavonoids and terpenoids [7,64,65], ethyl oleate’s binding profile appears less extensive in terms of direct interactions with the catalytic core.

The molecular docking analysis of ethyl oleate with the SARS-CoV-2 papain-like protease (PLpro) reveals significant interactions that suggest its potential as an antiviral agent. Ethyl oleate primarily forms van der Waals interactions with key residues such as Arg166, Gly163, Met206, and Leu162 (**Fig 2F**) within the PLpro binding pocket. Additionally, carbon hydrogen bonding occurs with Glu167 and Ser170, stabilizing the compound in the active site. Alkyl and Pi-alkyl interactions with Tyr264, Tyr268, and Tyr273 further contribute to hydrophobic stabilization [46]. These interactions indicate a moderate affinity of ethyl oleate for the PLpro active site, although its lack of direct interactions with the catalytic triad residues (Cys111, His272, and Asp286) may limit its inhibitory potential.

The docking analysis (**Fig 2G**) reveals several key interactions between the ligand and the target protein. The hydroxyl groups of 2,6-dihydroxybenzoic acid form hydrogen bonds with amino acid residues such as His41, Cys145, Leu141, and Ser144(**Fig 2G**) within the active site of Mpro [4,54,66]. The simultaneous engagement of the catalytic Cys145/His41 pair, the oxy-anion hole (Ser144), and the S1 sub-pocket (Leu141) in a single pose offers compelling structural validation of this compound as a high-quality pharmacophore.These interactions are crucial for the ligand’s binding and potential inhibition of the protease, which plays a central role in the viral replication process. Comparing these findings with recent literature, the observed binding interactions are consistent with previous computational studies exploring the antiviral potential of 2,6-dihydroxybenzoic acid against SARS-CoV-2 [9,58]. The compound’s ability to form hydrogen bonds with key catalytic residues in the Mpro active site suggests its potential as a lead compound for the development of COVID-19 therapeutic agents.

**Fig 2H** shows the results of a molecular docking simulation between 2,6-dihydroxybenzoic acid and the papain-like protease (PLpro) of SARS-CoV-2. The docking analysis reveals that 2,6-dihydroxybenzoic acid forms several hydrogen bond interactions with amino acid residues within the active site of PLpro. The hydroxyl groups of the ligand engage in hydrogen bonding with residues such as Thr301, Arg166, and Ser245 (**Fig 2H**), which are crucial for the enzyme’s catalytic function and viral replication. Interestingly, the docking results also indicate the formation of a Pi-alkyl interaction between the aromatic ring of the ligand and the side chain of Tyr273, as well as a Pi-Pi T-shaped interaction with Tyr268. These non-covalent interactions further stabilize the binding of the compound within the PLpro active site, potentially inhibiting the enzyme’s activity. The observed binding interactions are consistent with previous computational studies investigating the antiviral potential of natural compounds against SARS-CoV-2 [8,67]. The compound’s ability to form hydrogen bonds and other favorable interactions with key catalytic residues in the PLpro active site suggests its potential as a lead compound for the development of COVID-19 therapeutic agents.

### Study of the similarity of SARS-CoV-2 proteases with papain

#### Global alignment of PLpro and Mpro with papain using the EMBOSS Needle Algorithm.

The global alignment results between papain and the main protease (Mpro) of SARS-CoV-2, as generated by the EMBOSS needle program, provide valuable insights into the evolutionary relationships and functional similarities between these two proteolytic enzymes. The alignment indicates a total length of 486 amino acids with a low sequence identity of 9.7% (47/486), suggesting that while there may be some conserved regions, the overall sequences are quite divergent. The similarity score of 14.6% (71/486) further emphasizes this point, indicating that despite the low identity, there are segments of the proteins that exhibit functional relevance or structural conservation.

The high gap percentage of 66.0% (321/486) reveals significant insertions or deletions that have occurred over evolutionary time, which is common in homologous proteins that have adapted to different functions or substrates. The score of 22.0 reflects the overall quality of the alignment, which, while not particularly high, suggests that the conserved areas warrant further investigation. Notably, the alignment also highlights specific regions where the two sequences show conservation, which may be indicative of essential functional motifs.

These findings suggest that while papain and Mpro have diverged significantly, they may still retain certain functional similarities that could affect their biological roles. For instance, both enzymes are cysteine proteases, which play critical roles in physiological processes. Further structural and functional studies are warranted to explore the implications of these alignments, particularly in the context of drug design and therapeutic interventions targeting viral proteases [68,69].

The global alignment between papain and the papain-like protease (PLpro) of SARS-CoV-2, as performed using the EMBOSS needle program, reveals significant insights into the evolutionary and functional dynamics of these proteases (S2 Fig). The alignment encompasses a sequence length of 554 amino acids, yielding a low identity percentage of 6.1% (34/554) and a similarity percentage of 10.3% (57/554). These low values suggest considerable divergence between the two enzymes, which may reflect their adaptation to different biological functions and substrates. A striking feature of the alignment is the high gap percentage of 79.4% (440/554), indicating extensive insertions and deletions throughout the sequences. This high level of gaps is typical in comparisons of homologous proteins that have evolved under different selective pressures, potentially leading to the development of distinct catalytic properties. The alignment score of 31.5 suggests that, while the overall sequence conservation is minimal, there are segments where structural or functional similarities may still be relevant. The conserved regions, although limited, may correspond to critical functional motifs essential for the proteolytic activity of both enzymes. Understanding these conserved areas is crucial, particularly in the context of drug design, as inhibitors targeting PLpro could potentially be informed by the structural characteristics of papain [20,70]. Both enzymes are cysteine proteases, suggesting that despite their sequence divergence, they may share common catalytic mechanisms that warrant further investigation.

#### Multiple sequence alignment of PLpro and Mpro with papain.

The multiple sequence alignment of papain, the papain-like protease (PLpro), and the main protease (Mpro) of SARS-CoV-2, conducted using CLUSTAL Omega, reveals significant insights into the evolutionary relationships and functional conservation among these cysteine proteases (S3 Fig). The alignment highlights regions of sequence conservation and divergence, which are crucial for understanding their catalytic mechanisms and potential therapeutic targeting.

The alignment establishes a low sequence identity across the three proteases, with distinct conserved motifs that suggest functional importance. For instance, conserved residues are observed in critical regions associated with substrate binding and catalysis. The alignment also reveals extensive gaps, particularly in the sequences of PLpro and Mpro, indicating evolutionary adaptations that may reflect differing substrate specificities and biological roles. This is particularly relevant given that PLpro is known to play a role in viral replication and immune evasion by deubiquitinating host proteins, while Mpro is essential for processing the viral polyprotein into functional units [4,64].

The alignment includes notable regions where all three proteases exhibit significant conservation, such as the active site residues, which are critical for their proteolytic functions. The presence of similar catalytic residues suggests that, despite their low sequence identity, they may share analogous mechanisms of action. This conservation supports the hypothesis that these proteases, while evolving under different conditions, maintain essential functions necessary for their respective biological processes.

### Pharmacokinetics profiles (*in silico*) of the promising compounds

γ-Sitosterol, a phytosterol derived from *Moringa oleifera*, has potential as a SARS-CoV-2 protease inhibitor due to its intrinsic characteristics and potential as an antiviral drug (S4 Table). Its calculated log D7.4 (1.7) and high predicted human intestine absorption (71.29%) support oral administration, which is important for therapeutic use. The large volume of distribution (VDss 3.14 L/kg) suggests tissue penetration, which may help reach the targeted viral proteins in adequate concentrations.

Ethyl oleate has a pharmacokinetic profile suitable for SARS-CoV-2 topical or inhalation preparations (S4 Table). Due to its high lipophilicity (log KOW 6.59) and excellent projected tissue distribution (VDss 3.94 L/kg), the compound may cross cellular membranes and block viral multiplication. Low oral bioavailability (32.47%) is less important for a locally acting medication. The compound inhibits important cytochrome P450 enzymes, especially CYP2D6 (>100%), which may extend its effective half-life (71.3 hr) at the site of action by decreasing local metabolic clearance. The ethyl oleate’s favorable distribution and metabolic stability make it a promising locally-administered antiviral drug [13,46,71].

Pregn-5–7-diene-3-ol20-one, a steroid derivative, has promising ADMET for medicinal utilization (S4 Table). Its intermediate lipophilicity (log D7.4 = 1.67) and high projected intestinal absorption (65.17%) aid permeation, while low P-gp inhibition (31.33%) reduces efflux risk. The compound has good tissue penetration (VDss = 3.76 L/kg) and low blood-brain barrier crossing (17.04%), decreasing neurotoxicity concerns. It has substantial CYP2D6 inhibition (83.27%) and substrate activity (56.45%), which may give metabolic stability and a long half-life (56.18 hr) despite modest hepatic clearance. The profile recommends oral administration with a delivery system improvement [13,46,71].

### *In vitro* assessment of papain inhibition by *Moringa oleifera* leaf extracts

#### Phytochemical composition of *Moringa oleifera* leaves.

Table 3 presents the total polyphenol content of *Moringa oleifera* leaf extracts expressed in milligrams of gallic acid equivalent per gram of sample. The total polyphenol content of the aqueous extract of *Moringa oleifera* leaves is 145.12 ± 0.08 mg GAE/g. This content is significantly higher than that of the hydromethanolic extract (93.35 ± 0.23 mg GAE/g) (Table 3). These results are lower than those reported by Goel [72] who found 216.45 ± 4.6 mg GAE/g for the hydromethanolic extract and 88.5 ± 3.6 mg GAE/g for the aqueous extract. A very high value compared to the value found by Ghada *et al.*[73]. These variations in concentrations of phenolic compounds in plant species could be due to several reasons, including the presence of other secondary metabolites at different concentrations and other factors such as plant maturity and genotypes. It could also be due to extraction methods and different solvent polarities, storage duration, as well as different phytogeographical zones and plant harvesting seasons that can also alter the content of phenolic compounds and other phytochemical substances in these medicinal plants [46,74,75].

**Table 3:** Phytochemical composition and antioxidant properties of *Moringa oleifera’s* leaves.

Parameters	Extract		Reference	
	Aqueous	Hydromethanolic	Ascorbic acid	Quercetin
Total polyphenol (mg GAE/g)	145.12 ± 0.08ᵃ¹	93.35 ± 0.23^b^²	–	–
Flavonoid (mg QE/g)	95.86 ± 0.29^c^²	135.41 ± 0.37ᵃ¹	–	–
Tannin (mg CE/g)	53.65 ± 0.36^d^¹	35.16 ± 0.85ᵉ²	–	–
DPPH (mg TE/g DM)	123.58 ± 0.22^b3^	63.46 ± 0.02^c4^	140.12 ± 1.25^a2^	158.44 ± 1.12^a1^
FRAP (mg TE/g DM)	49.66 ± 0.023^e3^	48.14 ± 0.02^d4^	52.18 ± 0.94^b2^	56.77 ± 1.03^b1^

Results with the same numbers (1, 2, 3) on the same row are not significantly different (p > 0.05). Results with the same letters (a, b, c) on the same column are not significantly different (p > 0.05).

The flavonoid content of *Moringa oleifera* leaf extracts varies significantly at p < 0.05. The hydromethanolic extract has more significant flavonoid content, at 135.41 ± 0.37 mg QE/g (**Table 3**). This content is much higher than that of the aqueous extract (95.86 ± 0.29 mg QE/g). The results obtained are higher than those reported by Goel [72] which were 65.38 mg QE/g for the hydromethanolic extract and 83.21 ± 50 mg QE/g for the aqueous extract; and higher than the results reported by Ghada *et al.*[73] who found 57.64 mg QE/g for the hydromethanolic extract and 24.93 mg QE/g. These differences could be explained by the impact of extrinsic factors in the environment such as geographical location, soil nature, drought, stress, and diseases. According to Mamoudou *et al.* [11], flavonoid levels increase when the plant’s living environment is not adequate, in which case the plant promotes the synthesis of secondary metabolites to adapt and survive.

The tannin content of *Moringa oleifera* leaf extracts varies significantly at p < 0.05. The hydromethanolic extract has more significant tannin content, at 53.65 ± 0.36 mg CE/g (**Table 3**). This content is much higher than that of the aqueous extract (35.16 ± 0.85 mg CE/g). These results are lower than those reported by Ghada *et al.*[73], and also higher than the results reported by Mune Mune *et al.*[42] which were 16.54 mg CE/g for the aqueous extract and 29.08 mg CE/g for the hydromethanolic extract. This difference could be explained by the influence of genetic and geographical factors and the degree of plant maturation.

These findings suggest that *M. oleifera* leaves represent a promising source of natural compounds that warrant further exploration as potential SARS-CoV-2 protease inhibitors.

#### Antioxidant activity of *Moringa oleifera* leaves.

The role of antioxidants in mitigating inflammation and oxidative stress during viral infections, including COVID-19, has been widely documented [3,14,53]. Elevated pro-inflammatory cytokines and oxidative stress contribute to endothelial dysfunction, platelet activation, and disease severity. Accordingly, the antioxidant properties of *Moringa oleifera* leaf extracts were evaluated using the DPPH radical scavenging assay and the ferric reducing antioxidant power (FRAP) assay (Table 3).

The aqueous extract showed markedly higher DPPH scavenging activity (123.58 ± 0.22 mg TE/g DM) than the hydromethanolic extract (63.46 ± 0.02 mg TE/g DM). When compared to standard antioxidants, the aqueous extract demonstrated an activity approaching that of ascorbic acid (140.12 ± 1.25 mg TE/g DM) and quercetin (158.44 ± 1.12 mg TE/g DM). This remarkable similarity in DPPH scavenging capacity to pharmacological reference standards such as ascorbic acid (a principal antioxidant vitamin) and quercetin (a highly potent flavonoid standard) substantiates the significant free radical neutralizing ability of the Moringa extract. This indicates that the extract possesses strong radical scavenging capacity attributable to its high polyphenol (145.12 mg GAE/g) and tannin (53.65 mg CE/g) content. Little differences in these values were observed in the study of Ghada *et al.*[73]. Polyphenols and tannins in *Moringa oleifera* leaves probably contributed to the high DPPH scavenging activity of the aqueous extract. These two classes of phytochemicals play important role not only in the inhibition of key viral enzymes, but also in the regulation of the inflammatory response by the production of interferons, in addition to iron chelating activity which promoted hemoglobin stability and iron dysmetabolism during the viral infection [76–78].

Similarly, FRAP values of the aqueous (49.66 ± 0.02 mg TE/g DM) and hydromethanolic (48.14 ± 0.02 mg TE/g DM) extracts were comparable to ascorbic acid (52.18 ± 0.94 mg TE/g DM) and within the range of quercetin (56.77 ± 1.03 mg TE/g DM).The observed similarity in FRAP values shows that the Moringa extracts have a strong ability to donate electrons that is similar to these reference molecules. This confirms that they could be used to reduce pro-oxidant states effectively. These results also demonstrate that *M. oleifera* extracts exhibit ferric reducing power comparable to widely used reference antioxidants. These values were higher than those reported in other studies [42,73]. High ferric reducing power suggested *Moringa oleifera* leaves may play important role in maintaining important functions such as oxygen transport and energy production then prevent inflammation [14,79].

The quantitative comparison against the high-efficacy standards (ascorbic acid and quercetin) provides a strong foundation for the use of *M. oleifera*, confirming the extracts possess substantial antioxidant activity necessary to mitigate oxidative stress and preserve endothelial function during the inflammatory stages of viral infections.

### Kinetic study of the inhibition of papain by *Moringa oleifera* leaves extract

Papain, a cysteine protease, presented high similarity in the active site residues with SARS-CoV-2 proteases Mpro and PLpro and then probably similar molecular mechanism of the enzyme reaction. In instance, the active site of SARS-CoV-2 Mpro contains a catalytic dyad (Cys145/His41) and the one in papain contains a catalytic triad consisting of Cys25, His159 and Arg175 [7,26,59]. Therefore, papain was chosen for kinetic studies to substitute Mpro and PLpro. Inhibition of papain by aqueous and hydromethanolic extracts showed a dose-dependent trend with increasing percentage inhibition with the concentration of extracts. The half-maximum inhibition concentration (IC_50_) was lower for the hydromethanolic extract (7.5 mg/mL) compared to the aqueous extract (12.5 mg/mL). This was expectable since water extracted other important compounds in *Moringa oleifera* leaves such as proteins [18,42,80]. These findings are consistent with recent studies that have highlighted the potential of *Moringa oleifera* compounds, such as isothiocyanates and flavonoids, to inhibit cysteine proteases like Mpro [7,64].

**Fig 3:**
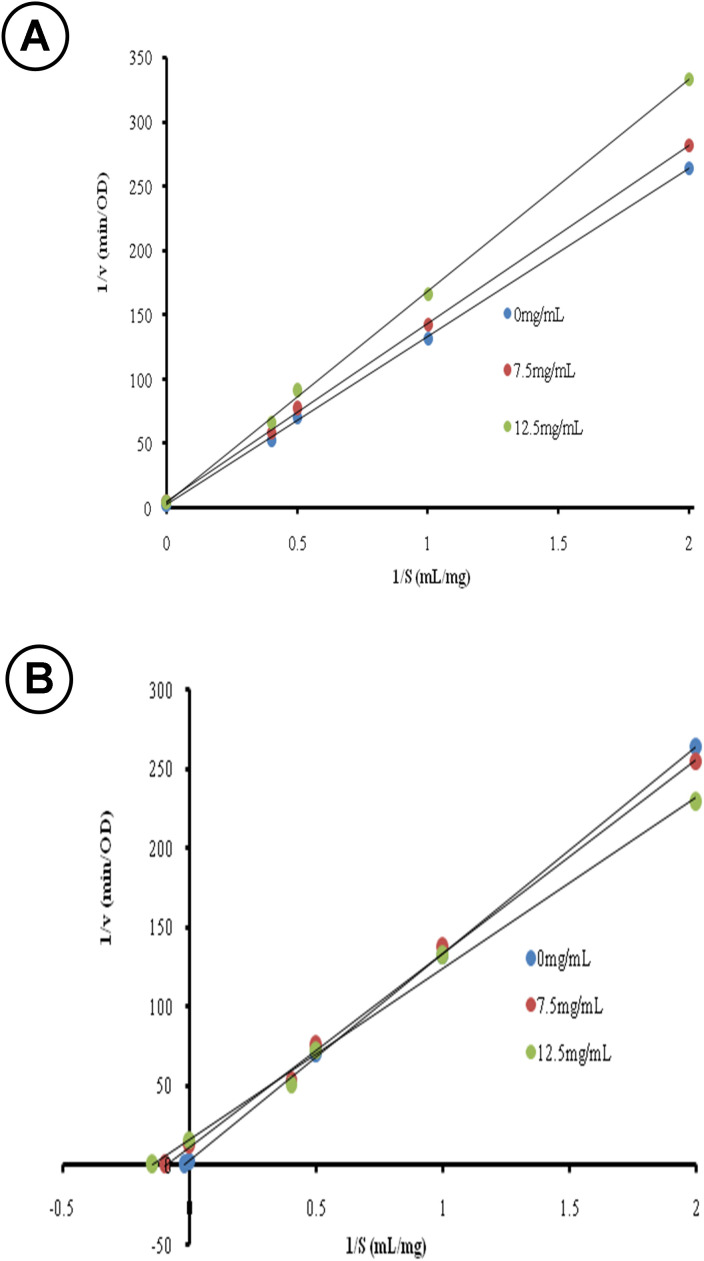
Lineweaver–Burk plot for the inhibition of papain. (A) Hydromethanolic extract. (B) Aqueous extract of *Moringa oleifera* leaves.

The Lineweaver-Burk plot was then constructed from the initial velocity data to define how compounds in extracts interact with the enzyme during the enzymatic reaction and to determine the kinetic parameters of each inhibitor (extract) [36]. Results probably represent a global inhibitory trend of the entire compounds present in the extract. It was observed that the Lineweaver-Burk plots for the hydromethanolic extract intercepted on the y-axis and the value of 1/Vmax remained constant (Table 4, Fig 3A) while the slope increased with the extract concentration. These results showed that hydromethanolic extract act as competitive inhibitors for papain. It could be assumed that compounds in this extract interacted with the amino acid residues at the active site of the enzyme. However, the interaction was not tight since the inhibition constant (Ki) was high (49.47 mg/mL). On another hand, the Lineweaver-Burk plot for the aqueous extract of *Moringa oleifera* leaves showed different trend (Fig 3B). The plots at different extract concentration interacted at the right part of the graph, with the slope decreasing with the increasing inhibitor concentration. Consequently, $V_{\text{max}}^{app}$ and $K_{\text{m}}^{app}$decreased with increasing inhibitor concentration.

**Table 4:** Kinetic parameters ($V_{max}^{app}$ and $K_{m}^{app}$) of the inhibition of papain, a cysteine-protease enzyme.

Inhibitor	$V_{max}^{app}$(DO/min)	$V_{max}^{app}$(DO/min)	$V_{max}^{app}$(DO/min)	$K_{m}^{app}$(mg/mL)	$K_{m}^{app}$(mg/mL)	$K_{m}^{app}$(mg/mL)	Inhibition type	*K*_*i*_(mg/mL)	K_*I*_(mg/mL)
	0	A	B	0	A	B			
H₂O	0.37	0.09	0.06	48.69	10.99	6.89	Hyperbolic non-competitive	50	2.7
MeOH	0.37	0.37	0.37	48.69	52.27	61.67	Competitive	49.47	–

Values are means of duplicate determination. The characters (A, B) represent the concentration of the inhibitor: A = 7.5 mg/mL and B = 12.5 mg/mL.

**Fig 4:**
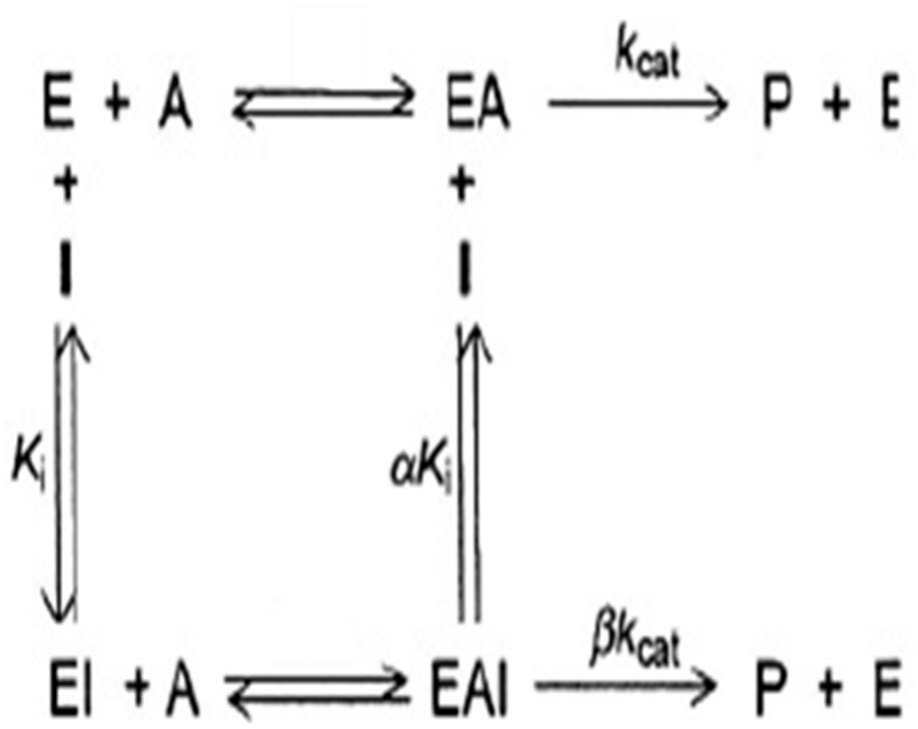
Interactions in hyperbolic non-competitive inhibition (E Enzyme, A substrate, I inhibitor, P product).

This trend is characteristic to hyperbolic non-competitive inhibition [36], in which the inhibitor interacts with the free enzyme, as well as the enzyme already bound to the substrate, and enzyme bound to the inhibitor preserves the capacity to interact with the substrate (Fig 4). The inhibitory constants Ki and K_I_ represent the strength of interaction between the inhibitor and E and ES, respectively. It could then be assumed that compounds in the hydromethanolic extract bound to E with weak interactions since Ki was high and similar to that of the aqueous extract. However, interaction of the inhibitor with ES was almost 20-times higher than the interaction with E (Table 4). Aqueous extract probably contained compounds with high affinity with the ES complex, which could be interesting for the potential treatment or prevention of COVID-19 disease.

### Constraints of papain as a surrogate protease

Papain’s catalytic Cys–His chemistry makes it a valuable and informative early biochemical screen, although it cannot replace SARS-CoV-2 Mpro and PLpro. Papain, a plant clan-CA enzyme, differs from viral Mpro, a 3C-like protease with a Cys–His dyad, and PLpro, a big multi-domain deubiquitinating protease. Papain and the viral proteases have low sequence identity and large gap fractions in global alignments, implying significant fold and substrate-binding architectural divergence. Thus, drugs that block papain may interact differently with Mpro and PLpro’s binding pockets, substrate specificities, and conformational dynamics. Papain inhibition is only evidence that an extract or compound can affect cysteine protease chemistry *in vitro*, not antiviral action.

## Conclusion

This study provides computational and biochemical evidence that *Moringa oleifera* leaves contain bioactive compounds with potential for the treatment and prevention of COVID-19 disease. Molecular docking analysis identified γ-sitosterol as the most promising compound for the inhibition of the SARS-CoV-2 main protease (Mpro) and papain-like protease (PLpro). Moreover, hydromethanolic and aqueous extracts from *Moringa oleifera* leaves exhibited a cysteine-protease inhibitory activity in a papain-based assay, and kinetic analysis revealed competitive and hyperbolic non-competitive inhibition mechanisms, respectively. The extracts also presented notable antioxidant activity, which correlated with their high quantity of polyphenols and flavonoids. The findings indicate that *M. oleifera* leaves possess phytochemicals that exhibit favorable docking poses to SARS-CoV-2 Mpro and PLpro and inhibit a model cysteine protease (papain) *in vitro*. These findings collectively justify the prioritization of specific compounds for further investigation, including direct biochemical inhibition assays against purified recombinant Mpro and PLpro, biophysical binding validation, counterscreening for promiscuous cysteine reactivity, and cell-based antiviral and cytotoxicity assays. Until validation is completed, the inhibition of papain should be regarded as a preliminary screening result rather than direct evidence of therapeutic efficacy against COVID-19.

## Supporting information

S1 Fig Chemical structure of phytochemicals from *Moringa oleifera* leaves: (A) 1,2,3-Cyclopentanetriol, (B) 2,6-Dihydroxybenzoic acid, (C) Ethyl oleate, (D) Gamma-sitosterol, (E) Hexadecanal, (F) Octadecanoic acid, (G) Oleic acid, (H) Phytol, (I) Pregn-5,7-diene-3-ol20-one, (J) Quinic acid, (K) Vitamin E (L) cis-vaccenic acid, (M) L-galactose, 6-deoxy, (N) n-Hexadecanoic acid. (TIF)

S2 Fig Global alignment between papain and the papain-like protease (PLPro). (DOCX)

S3 Fig Multiple sequence alignment of PL^pro^ and M^pro^ with papain using Clustal Omega. (DOCX)

S4 Table ADMET properties of γ-sitosterol. Ethyl-oleate and Pregn-5–7-diene-3-ol20-one.
(DOCX)

## Abbreviations

Mpro: Main Protease
PLpro: Papain-Like Protease
