# Supplementary figures and images for: Moringa oleifera potential for the treatment and prevention of COVID-19 involving molecular interaction, antioxidant properties and kinetic mechanism
Source: PLoS One. 2025 Dec 3;20(12):e0337904. doi: 10.1371/journal.pone.0337904 (PMC12674540; doi:10.1371/journal.pone.0337904)

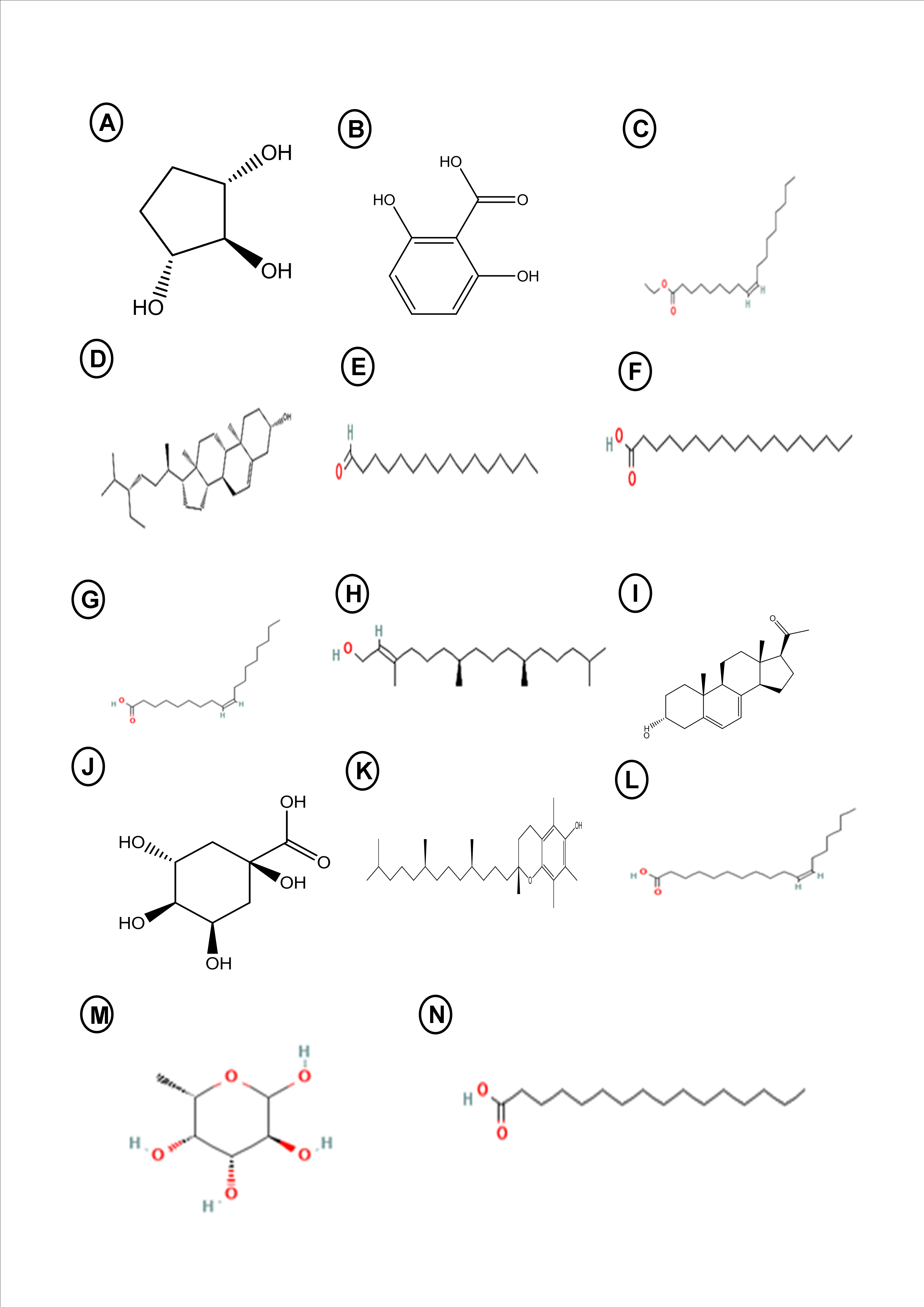

Supplement: S1 Fig — (TIF) [file pone.0337904.s001.tif]
