# Supplementary material for: Moringa oleifera potential for the treatment and prevention of COVID-19 involving molecular interaction, antioxidant properties and kinetic mechanism
Source: PLoS One. 2025 Dec 3;20(12):e0337904. doi: 10.1371/journal.pone.0337904 (PMC12674540; doi:10.1371/journal.pone.0337904)
Supplement: S2 Fig — (DOCX) [file pone.0337904.s002.docx]

**S2 Fig**

| ########################################  # Program: needle  # Rundate: Sat 23 Dec 2023 15:09:01  # Commandline: needle  # -auto  # -stdout  # -asequence emboss_needle-I20231223-150856-0521-4849222-p1m.aupfile  # -bsequence emboss_needle-I20231223-150856-0521-4849222-p1m.bupfile  # -datafile EBLOSUM62  # -gapopen 10.0  # -gapextend 0.5  # -endopen 10.0  # -endextend 0.5  # -aformat3 pair  # -sprotein1  # -sprotein2  # Align_format: pair  # Report_file:stdout  ########################################  #=======================================  # Aligned_sequences: 2  # 1: 6LU7A  # 2: PAPA1_CARPA  # Matrix: EBLOSUM62  # Gap_penalty: 10.0  # Extend_penalty: 0.5  #  # Length: 486  # Identity: 47/486 (9.7%)  # Similarity: 71/486 (14.6%)  # Gaps: 321/486 (66.0%)  # Score: 22.0  #=======================================  6LU7A 1 -------------------------------------------------- 0  PAPA1_CARPA 1 MAMIPSISKLLFVAICLFVYMGLSFGDFSIVGYSQNDLTSTERLIQLFES 50  6LU7A 1 -------------------------------------------------- 0  PAPA1_CARPA 51 WMLKHNKIYKNIDEKIYRFEIFKDNLKYIDETNKKNNSYWLGLNVFADMS 100  6LU7A 1 -------------------------------------SGFRKMAFPSGKV 13  ..:\|: .\|.\|  PAPA1_CARPA 101 NDEFKEKYTGSIAGNYTTTELSYEEVLNDGDVNIPEYVDWRQ----KGAV 146  6LU7A 14 EGCMVQVTCGTTTLNGLWLDDVVYCPRHVICTSEDMLNPNYEDLLIRKSN 63  .....\|.:\|\|: .\|....\|.....:\|......\|\|...\|..\|:....  PAPA1_CARPA 147 TPVKNQGSCGS-----CWAFSAVVTIEGIIKIRTGNLNEYSEQELLDCDR 191  6LU7A 64 HNFLVQAG----NVQL-RVIGHSMQN----------CVLKLKVDTANPKT 98  .::....\| .:\|\| ...\|...:\| \|..:.\| .\|..  PAPA1_CARPA 192 RSYGCNGGYPWSALQLVAQYGIHYRNTYPYEGVQRYCRSREK----GPYA 237  6LU7A 99 PKYKFVR-IQPGQTFSVLACYNGSPSGVYQCAMRPNFTI-KGSFLNGSCG 146  .\|...\|\| :\|\|....::\|......\|..\|...\|...:\|.: :\|....\|.\|\|  PAPA1_CARPA 238 AKTDGVRQVQPYNEGALLYSIANQPVSVVLEAAGKDFQLYRGGIFVGPCG 287  6LU7A 147 --------SVGFNIDYDCVSFCYMHHMELPTG--------VHAGTDLEGN 180  :\|\|:..:\|..:...: .\|\| :..\|\| \|\|  PAPA1_CARPA 288 NKVDHAVAAVGYGPNYILIKNSW------GTGWGENGYIRIKRGT---GN 328  6LU7A 181 FYG----------PFVDRQTAQAAGTDTTITVNVLAWLYAAVINGDRWFL 220  .\|\| \|..:  PAPA1_CARPA 329 SYGVCGLYTSSFYPVKN--------------------------------- 345  6LU7A 221 NRFTTTLNDFNLVAMKYNYEPLTQDHVDILGPLSAQTGIAVLDMCASLKE 270  PAPA1_CARPA 346 -------------------------------------------------- 345  6LU7A 271 LLQNGMNGRTILGSALLEDEFTPFDVVRQCSGVTFQ 306  PAPA1_CARPA 346 ------------------------------------ 345  #---------------------------------------  #--------------------------------------- |
| --- |

**S2 Fig.** Global alignment between papain and the papain-like protease (PLPro)
