# Supplementary material for: Moringa oleifera potential for the treatment and prevention of COVID-19 involving molecular interaction, antioxidant properties and kinetic mechanism
Source: PLoS One. 2025 Dec 3;20(12):e0337904. doi: 10.1371/journal.pone.0337904 (PMC12674540; doi:10.1371/journal.pone.0337904)
Supplement: S3 Fig — (DOCX) [file pone.0337904.s003.docx]

**S3 Fig**


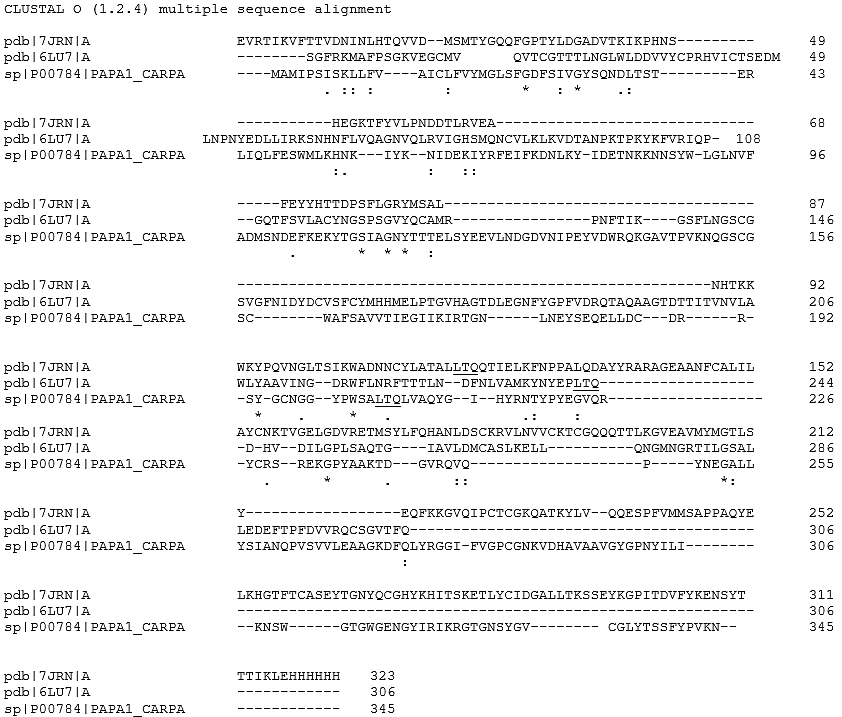


**S3 Fig.** Multiple sequence alignment of PL^pro^ and M^pro^ with papain using Clustal Omega application
