# Supplementary material for: Moringa oleifera potential for the treatment and prevention of COVID-19 involving molecular interaction, antioxidant properties and kinetic mechanism
Source: PLoS One. 2025 Dec 3;20(12):e0337904. doi: 10.1371/journal.pone.0337904 (PMC12674540; doi:10.1371/journal.pone.0337904)
Supplement: S4 Table — Ethyl-oleate and Pregn-5–7-diene-3-ol20-one. (DOCX) [file pone.0337904.s004.docx]

S4 Table. ADMET properties of γ-sitosterol

| Compound | γ-sitosterol |  |  |
| --- | --- | --- | --- |
| Molecule Property | Value | Unit | Reference |
| Molecular Weight | 414.39 | g/mol | Optimal |
| Number of Heteroatoms | 1 | / | Optimal |
| Number of Rotatable Bonds | 6 | / | Optimal |
| Number of Rings | 4 | / | Optimal |
| Number of HA | 1 | / | Optimal |
| Number of HD | 1 | / | Optimal |
| log KOW | 8.02 | log-ratio | Optimal |
| Absorption | Prediction | Unit | Reference |
| Caco-2 Permeability | -5.18 | log(cm/s) | Data / Optimal |
| HIA | 71.29 | % | Data / Optimal |
| Pgp Inhibition | 28.68 | % | Data / Optimal |
| log D7.4 | 1.7 | log-ratio | Data / Optimal |
| Aqeuous Solubility | -4.02 | log(mol/L) | Data / Optimal |
| Oral Bioavailability | 38.61 | % | Data |
| Distribution | Prediction | Unit | Reference |
| BBB | 17.8 | % | Data / Optimal |
| PPBR | 44.67 | % | Data / Optimal |
| VDss | 3.14 | L/kg | Data / Optimal |
| Metabolism | Prediction | Unit | Reference |
| CYP2C9 Inhibition | 48.34 | % | Data |
| CYP2D6 Inhibition | 91.74 | % | Data |
| CYP3A4 Inhibition | 33 | % | Data |
| CYP2C9 Substrate | 31.34 | % | Data |
| CYP2D6 Substrate | 48.43 | % | Data |
| CYP3A4 Substrate | 31.7 | % | Data |
| Excretion | Prediction | Unit | Reference |
| Half Life | 58.54 | hr | Data |
| CL-Hepa | 50.83 | uL min^-1^ (10^6^ cells)^-1^ | Data |
| CL-Micro | 37.23 | mL min^-1^ g^-1^ | Data |
| Toxicity | Prediction | Unit | Reference |
| hERG Blockers | 43.87 | % | Data / Optimal |
| Ames | 39.1 | % | Data / Optimal |
| DILI | 52.66 | % | Data / Optimal |
| LD50 | 2.47 | -log(mol/kg) | Data |

S4Table (continue). ADMET properties ofEthyl-oleate

| Compound | Ethyl-oleate |  |  |
| --- | --- | --- | --- |
| Molecule Property | Value | Unit | Reference |
| Molecular Weight | 310.29 | g/mol | Optimal |
| Number of Heteroatoms | 2 | / | Optimal |
| Number of Rotatable Bonds | 16 | / | Optimal |
| Number of Rings | 0 | / | Optimal |
| Number of HA | 2 | / | Optimal |
| Number of HD | 0 | / | Optimal |
| log KOW | 6.59 | log-ratio | Optimal |
| Absorption | Prediction | Unit | Reference |
| Caco-2 Permeability | -5.1 | log(cm/s) | Data / Optimal |
| HIA | 60.8 | % | Data / Optimal |
| Pgp Inhibition | 41.24 | % | Data / Optimal |
| log D7.4 | 1.73 | log-ratio | Data / Optimal |
| Aqeuous Solubility | -4.84 | log(mol/L) | Data / Optimal |
| Oral Bioavailability | 32.47 | % | Data |
| Distribution | Prediction | Unit | Reference |
| BBB | 35.83 | % | Data / Optimal |
| PPBR | 54.67 | % | Data / Optimal |
| VDss | 3.94 | L/kg | Data / Optimal |
| Metabolism | Prediction | Unit | Reference |
| CYP2C9 Inhibition | 74.11 | % | Data |
| CYP2D6 Inhibition | 101.62 | % | Data |
| CYP3A4 Inhibition | 36.3 | % | Data |
| CYP2C9 Substrate | 32.49 | % | Data |
| CYP2D6 Substrate | 43.47 | % | Data |
| CYP3A4 Substrate | 31.99 | % | Data |
| Excretion | Prediction | Unit | Reference |
| Half Life | 71.3 | hr | Data |
| CL-Hepa | 58.6 | uL min^-1^ (10^6^ cells)^-1^ | Data |
| CL-Micro | 38.9 | mL min^-1^ g^-1^ | Data |
| Toxicity | Prediction | Unit | Reference |
| hERG Blockers | 42.37 | % | Data / Optimal |
| Ames | 35.42 | % | Data / Optimal |
| DILI | 51.54 | % | Data / Optimal |
| LD50 | 1.25 | -log(mol/kg) | Data |

S4 Table (continue).. ADMET properties ofPregn-5-7-diene-3-ol20-one

| Compound | Characteristics | Value | Unit | Reference |
| --- | --- | --- | --- | --- |
| *Pregn-5-7-diene-3-ol20-one* | |  |  |  |
|  | Molecule Property |  |  |  |
|  | Molecular Weight | 334.25 | g/mol | Optimal |
|  | Number of Heteroatoms | 3 | / | Optimal |
|  | Number of Rotatable Bonds | 1 | / | Optimal |
|  | Number of Rings | 4 | / | Optimal |
|  | Number of HA | 3 | / | Optimal |
|  | Number of HD | 2 | / | Optimal |
|  | log KOW | 3.71 | log-ratio | Optimal |
|  | Absorption | Prediction | Unit | Reference |
|  | Caco-2 Permeability | -5.26 | log(cm/s) | Data / Optimal |
|  | HIA | 65.17 | % | Data / Optimal |
|  | Pgp Inhibition | 31.33 | % | Data / Optimal |
|  | log D7.4 | 1.67 | log-ratio | Data / Optimal |
|  | Aqeuous Solubility | -4.31 | log(mol/L) | Data / Optimal |
|  | Oral Bioavailability | 41.97 | % | Data |
|  | Distribution | Prediction | Unit | Reference |
|  | BBB | 17.04 | % | Data / Optimal |
|  | PPBR | 46.6 | % | Data / Optimal |
|  | VDss | 3.76 | L/kg | Data / Optimal |
|  | Metabolism | Prediction | Unit | Reference |
|  | CYP2C9 Inhibition | 51.77 | % | Data |
|  | CYP2D6 Inhibition | 83.27 | % | Data |
|  | CYP3A4 Inhibition | 30.83 | % | Data |
|  | CYP2C9 Substrate | 28.49 | % | Data |
|  | CYP2D6 Substrate | 56.45 | % | Data |
|  | CYP3A4 Substrate | 28.3 | % | Data |
|  | Excretion | Prediction | Unit | Reference |
|  | Half Life | 56.18 | hr | Data |
|  | CL-Hepa | 46.28 | uL min^-1^(10^6^ cells)^-1^ | Data |
|  | CL-Micro | 33.34 | mL min^-1^ g^-1^ | Data |
|  | Toxicity | Prediction | Unit | Reference |
|  | hERG Blockers | 40.6 | % | Data / Optimal |
|  | Ames | 41.78 | % | Data / Optimal |
|  | DILI | 46.14 | % | Data / Optimal |
|  | LD50 | 2.2 | -log(mol/kg) | Data |
